# Chronic inflammatory diseases are stimulated by current lifestyle: how diet, stress levels and medication prevent our body from recovering

**DOI:** 10.1186/1743-7075-9-32

**Published:** 2012-04-17

**Authors:** Margarethe M Bosma-den Boer, Marie-Louise van Wetten, Leo Pruimboom

**Affiliations:** 1 University of Girona, Plaça Sant Domènec, 3 Edifici Les Àligues, 17071, Girona, Spain

**Keywords:** Chronic inflammation, Central stress system, Nutrition, Resoleomics, Sympathetic-adrenal-medulla axis, Hypothalamus-pituitary-adrenal axis, Anti-inflammatory medication, Insulin resistance, Polyunsaturated fatty acids, Glycemic index

## Abstract

Serhan and colleagues introduced the term "Resoleomics" in 1996 as the process of inflammation resolution. The major discovery of Serhan's work is that onset to conclusion of an inflammation is a controlled process of the immune system (IS) and not simply the consequence of an extinguished or "exhausted" immune reaction. Resoleomics can be considered as the evolutionary mechanism of restoring homeostatic balances after injury, inflammation and infection. Under normal circumstances, Resoleomics should be able to conclude inflammatory responses. Considering the modern pandemic increase of chronic medical and psychiatric illnesses involving chronic inflammation, it has become apparent that Resoleomics is not fulfilling its potential resolving capacity. We suggest that recent drastic changes in lifestyle, including diet and psycho-emotional stress, are responsible for inflammation and for disturbances in Resoleomics. In addition, current interventions, like chronic use of anti-inflammatory medication, suppress Resoleomics. These new lifestyle factors, including the use of medication, should be considered health hazards, as they are capable of long-term or chronic activation of the central stress axes. The IS is designed to produce solutions for fast, intensive hazards, not to cope with long-term, chronic stimulation. The never-ending stress factors of recent lifestyle changes have pushed the IS and the central stress system into a constant state of activity, leading to chronically unresolved inflammation and increased vulnerability for chronic disease. Our hypothesis is that modern diet, increased psycho-emotional stress and chronic use of anti-inflammatory medication disrupt the natural process of inflammation resolution ie Resoleomics.

## Introduction

The number of people suffering from chronic diseases such as cardiovascular diseases (CVD), diabetes, respiratory diseases, mental disorders, autoimmune diseases (AID) and cancers has increased dramatically over the last three decades. The increasing rates of these chronic systemic illnesses suggest that inflammation [1,2], caused by excessive and inappropriate innate immune system (IIS) activity, is unable to respond appropriately to danger signals that are new in the context of evolution. This leads to unresolved or chronic inflammatory activation in the body.

Inflammation is designed to limit invasions and damage after injury, a process which has been essential for the survival of Homo sapiens in the absence of medication such as antibiotics. Recently, it has been discovered that onset to conclusion of an inflammation is a self-limiting and controlled process of the immune system (IS). This process of inflammation resolution is defined by Serhan as Resoleomics [3], a term which will be used throughout this article.

Our genes and physiology, which are still almost identical to those of our hunter-gatherer ancestors of 100,000 years ago, preserve core regulation and recovery processes [4,5]. Nowadays our genes operate in an environment which is completely different to the one for which they were designed.

Modern man is exposed to an environment which has changed enormously since the time of the industrial revolution. In recent decades there has been a tremendous acceleration in innovations which have changed our lives completely. As a consequence, more than 75% of humans do not meet the minimum requirement of the estimated necessary daily physical activity [6], 72% of modern food types is new in human evolution [7], psycho-emotional stress has increased and man is exposed to an overwhelming amount of information on a daily basis. All these factors combine to produce an environment full of modern danger signals which continuously activate the IIS and central stress axes. The question is whether the IIS and its natural inflammatory response, Resoleomics, can still function optimally in this modern, fast-changing environment, considering that the IIS is designed to produce short, intensive reactions to acute external danger [8,9]. It would seem that in the bodies of people who have adopted a Western lifestyle the inflammatory response is not concluded because of an initial excessive or subnormal onset of the response [10].

This article postulates how triggers from chronic altered diet and psycho-emotional stress negatively influence Resoleomics, thereby increasing susceptibility to the development of chronic, low-grade, inflammation-based diseases due to the constant activation of both the central stress axes and the IIS. In addition, an attempt is made to demonstrate the ways in which the use of anti-inflammatory medication could influence Resoleomics.

## Resoleomics, a self-limiting process of inflammation

**Figure 1:**
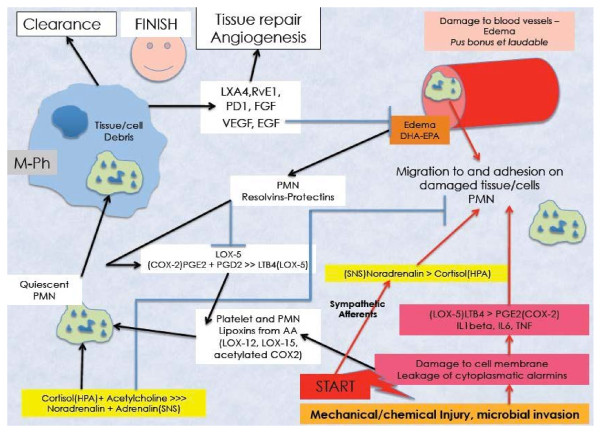
**Start and finish of a physiological inflammatory reaction in wound healing and situations of microbial challenge**. Cellular damage and leakage of alarmins attract neutrophils to the damaged area (PMN's). Sympathetic afferents activate the locus coeruleus (central nucleus of the sympathetic nervous system, SNS) and Noradrenaline (Norepinephrine, NE) is released. The released NE activates the adrenal medulla inducing the production of systemic catecholamins that supports the activation of the PMN. Damaged blood vessels are a source of an omega 3 rich edema (EPA and DHA). DHA and EPA inhibit LOX-5 directly and through conversion into resolvins and protectins. Both PGE2 and PGD2, produced by the breakdown of AA by COX-2 activity, will now override the strong chemotaxic effect of LTB4. The combined action of protectins, resolvins and lipoxins produced out of AA will put a hold on the pro-inflammatory activity of PMN's, which is supported by the increased production of systemic cortisol. Cortisol further activates macrophages (M-Ph) to phagocytose issue debris and quiet PMN by releasing substances such as LXA4, resolvin E1 (RvE1), prostanoid D1 (PD1), fibroblast growth factor (FGF), vascular endothelial growth factor (VEGF) and epithelial growth factor (EGF) at the same time. Further edema leakage will be stopped, whereas angiogenesis and production of connective tissue will take place, finishing the inflammatory reaction and starting the production of new tissue.

**Figure 2:**
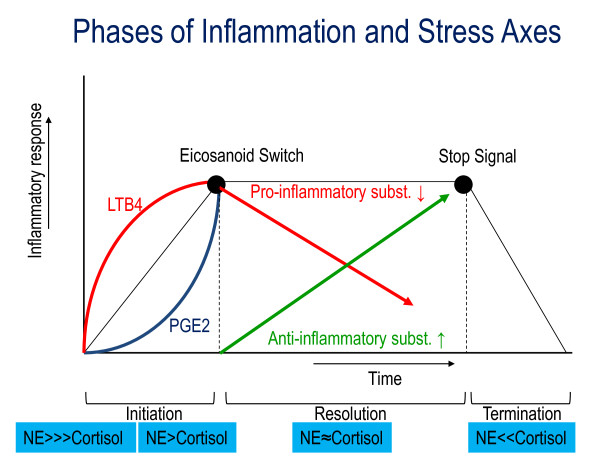
**Inflammation is a controlled process with an initiation, resolution and termination phase**. After microbial invasion, lesion or chemical injury, the initiation phase starts with the production of pro-inflammatory mediators like LTB4 and PG2. These mediators increase inflammation until the Eicosanoid Switch, the end of the initiation phase, takes place. This occurs when the level of PGE2 plus PGD2 is equal to the LTB4 level. The resolution phase is entered, triggering the generation of anti-inflammatory mediators like LK, resolvins, protectins, maresins, PGD2 and PGF2a. When the total level of anti-inflammatory mediators exceeds the level of LTB4 the Stop Signal takes place. This is the last phase, the inflammation will be terminated by clearing the affected area [11]. The stress hormones produced by the systemic stress axes have a direct effect on the inflammation phases. A microbial invasion, lesion or injury sends off an alarm in the body, setting off the systemic stress system which produces NE as response and tunes the system to insulin and cortisol resistance [12]. The Eicosanoids Switch to resolution can only take place when NE is equal to the level of cortisol plus insulin and when cortisol sensitivity is recovered. The Stop Signal requires a low level of NE and normalized cortisol sensitivity. The termination phase is entered when the stress axes are switched off.

Serhan and his colleagues [3] introduced the term Resoleomics to describe a self-limiting process of inflammation, executed and controlled by the innate immune system (IIS) and regulated by the sympathetic nervous system (SNS) and the hypothalamus-pituitary-adrenal (HPA) axis. This process controls inflammation using metabolites produced from arachidonic acid (AA), eicosapentaenoic acid (EPA) and docosahexenoic acid (DHA). Resoleomics operates locally when polymorphonuclear neutrophils (PMNs) are attracted by increased pro-inflammatory cytokine and eicosanoids production during microbial invasion, wound healing or chemical injury. The function is to limit the inflammation response. The central control system of the inflammatory reaction is very complex. Local and central processes influence each other and both are responsible for an optimal resolving response (Figure 1). The local process can be divided into three phases [11] (Figure 2):

1. Initiation phase

2. Resolution phase

3. Termination phase

### Initiation phase

Pro-inflammatory eicosanoids, like leukotrienes B4 (LTB4) and prostaglandins (PGs) initiate the inflammatory response. PMNs generate LTB4 and PGE2 from precursor AA with the use of lipoxygenase-5 (LOX-5) and cyclo-oxygenase 2 (COX-2). Both eicosanoids enhance inflammation, LTB4 being the strongest chemotoxic compound of cytotoxic neutrophils. PGE2 and/or PGD2, although initially pro-inflammatory, determine the switch to the next phase, the resolution of the inflammation.

### Resolution phase

This phase starts with the Eicosanoid Switch to resolution. When the PGE2 and/or PGD2 level is equal to the level of LTB4, the PMNs activate the switch from pro-inflammatory to anti-inflammatory eicosanoids production by limiting the production of LOX-5. This switch is responsible for the production of anti-inflammatory lipoxins (LXs) from AA through activation of lipoxygenase -12 (LOX-12), lipoxygenase-15 (LOX-15) and acetylated COX-2 [13,14]. This last mechanism has been found to be responsible for the production of more stable aspirin-triggered LXs (ATLs) with a longer half-value period [15]. Other resolving metabolites that support LXs are resolvins, (neuro)protectins and maresins produced from respectively EPA and DHA [11,16]. A second substantial increase of COX-2 activity will produce anti-inflammatory PGs (PGD2 and PGF2a) during this phase [17].

### Termination phase

This phase starts when the Stop Signal takes place. This happens when sufficient anti-inflammatory mediators such as LXs are available to stop the pro-inflammatory process [13,14]. LXs are capable of inhibiting both PMN infiltration and the activity of cytotoxic cells of the ISS, inducing phagocytosis to clear debris by non-cytoxic macrophages and attenuating an accumulation of the pro-inflammatory transcription factors, ie nuclear factor-kappaB (NF-kB) and activator protein 1 (AP-1) [18,19].

## Central stress axes and Resoleomics

This section deals solely with the effect of the sympathetic, parasympathetic and the HPA axis on Resoleomics. The systemic stress system is closely linked to the IIS via the stress axes of our body. Anything that can activate the sympathetic-adrenal-medulla (SAM) and HPA axes will have its effect on the IIS [20] and therefore on Resoleomics. Seen in reverse, it is precisely the IIS that can trigger stress axes, inducing a systemic stress reaction in the body [21]. In the SNS, which initially activates the IIS, inhibition of the IIS is provided by the strong anti-inflammatory neurotransmitter acetylcholine (ACh), produced by the parasympathetic nervous system [22].

The systemic stress reaction follows a two-wave pattern. Activation of the SAM axis is considered the first wave, giving rise to the excretion of brain norepinephrine (NE) by the Locus Coeruleus (LC). The descending pathway activates sympathetic motor neurons in the medulla oblongata, which stimulate the adrenal glands (through sympathetic efferent nerves). The adrenal gland will now excrete catecholamines, which activate and induce proliferation of ISS cells. NF-kB increases pro-inflammatory cytokines production, such as interleukin 1-beta (IL1-β), interleukin 6 (IL-6) and tumor necrosis factor (TNF). Both the IIS and Th1 of the adaptive IS contain receptors sensitive to catecholamines. Cerebral catecholamines affect the activity of spleen, thymus, bone marrow and lymphoid nodes [23]. NE has been shown to activate the IIS at the onset of inflammation, while long-term activation of the SNS induces IIS inhibition [24].

The second wave of the systemic stress reaction corresponds with the activation of the HPA axis, with glucocorticoids (GCs) as end product. Cortisol is capable of inhibiting the IIS through the upward regulation of inhibiting factor kappa B (IkB), while informing the immunological cortex through the migration of different immune cells to the brain [25,26]. Cortisol, the regulator of the IIS response, can guide the inflammation into resolution phase. Termination is instigated when cortisol "overrules" the NE effect on NF-kB signalling through genetic influence and reduction of transcription of the NF-kB sensitive pro-inflammatory gene, resulting in the finalization of the inflammatory response (Figure 2).

This "termination" effect of cortisol is normally supported by a compensatory anti-inflammatory response through activation of the vagal anti-inflammatory loop [27]. The resulting production of ACh inhibits the IS through the alfa-7-nicotin-Acetylcholinergic Receptor (α7nAChR) [28] (Figure 1).

The SNS (NE) increases the initial pro-inflammatory immune response in the initiation phase, whereas delayed cortisol response, induced by the HPA axis, inhibits the pro-inflammatory response [29]. Integrity of the SAM axis with its NE response/reaction is necessary for an adequate initial inflammatory response [30]. At the beginning of the initiation phase, there is resistance to both cortisol and insulin in order to allow for the activation of the IIS [12]. At the end of this phase, cortisol sensitivity and insulin sensitivity should be recovered to facilitate the Eicosanoid Switch to the resolution phase.

Chronic stress exposure reduces the capacity to mount an acute stress response [31], resulting in an inadequate pro-inflammatory response. Chronic (psycho-emotional) stress situations can be responsible for the continuous production of catecholamines by the SAM axis. People suffering from "perpetual stress", for example the parents of a child with cancer, showed chronic, increased levels of circulating pro-inflammatory cytokines [26]. This situation requires a high level of energy expenditure. The metabolic rate is increased to provide extra energy for the brain (arousal of all senses), the heart muscle and the locomotive system. The existing cells from the IIS are activated and will proliferate (relatively low energy expenditure), whereas proliferation of new immune cells (much more costly energy expenditure) will be blocked. Further consequences of chronic SAM activity are narrowing of the cell spectrum of the IIS and complete loss of activity of the Th1 section of the adaptive IS, leading to an insufficient capability to fight viruses, (pre)neoplastic cells and intracellularly presented pathogens [31].

An inflammatory response leading to solution depends on the sensitivity of glucocorticoid receptors (GR) and catecholamine receptors of the IIS [32]. Factors such as stress endured early in life, trauma and polymorphisms are possible risk factors for loss of GR and catecholamine sensitivity [33-35].

Suboptimal inflammatory response as a consequence of chronic stress prevents the Eicosanoid Switch from functioning, since the switch to the resolution phase requires recovered cortisol and insulin sensitivity. The initiation phase should have a maximum duration of 8 to 12 hrs. PMN number and activation levels should reach their maximum during this phase; longer duration caused by chronic stress could produce secondary damage to neighbouring tissues due to the strong cytotoxic effects of activated PMNs [11]. Supramaximal activation of PMNs could sensitize the adapted IS if contact time between self-antigens and the IS is significantly increased [11,29].

The crosstalk between the IS and stress axes is further evidenced by the fact that acute production of high levels of catecholamines activate the IIS strongly [23], whereas eicosanoids produced from AA induce the production of local and systemic catecholamines [36]. Long-term activation may lead to catecholamine resistance and lack of eicosanoid production. This situation, combined with the aforementioned possibility of resistance to insulin and cortisol, provokes a suboptimal inflammatory response and consequently the perpetuation and development of low-grade inflammation [26,37].

## Nutritional factors and Resoleomics

Several dietary factors influence the activity of the IIS and the function of a wide range of hormones, including cortisol, insulin and catecholamines. The dramatic changes in dietary composition since the agricultural revolution (some 10,000 years ago) and, to a greater extent, since the industrial revolution (some 200 years ago) have turned the intake of food into a common daily danger and therefore a cause of continuous systemic stress. Some of these changes include an increase in the omega 6/omega 3 fatty acid ratio, a high intake of saturated fatty acids (SFA) and refined carbohydrates, the introduction of industrially produced trans fatty acids, a lower intake of vitamins D and K, imbalanced intake of antioxidants, high intake of anti-nutrients (eg lectines, saponins) and an altered intake of dietary fibre [38].

The following section will discuss the impact of the changed ratio of polyunsaturated fatty acids (PUFAs) and the intake of food with a high glycemic load on Resoleomics. The pro-inflammatory effects of anti-nutrients present in cereals [39], potatoes [40], legumes [41], and tomato have previously been extensively reviewed [7].

### Role of PUFAs in inflammation

**Figure 3:**
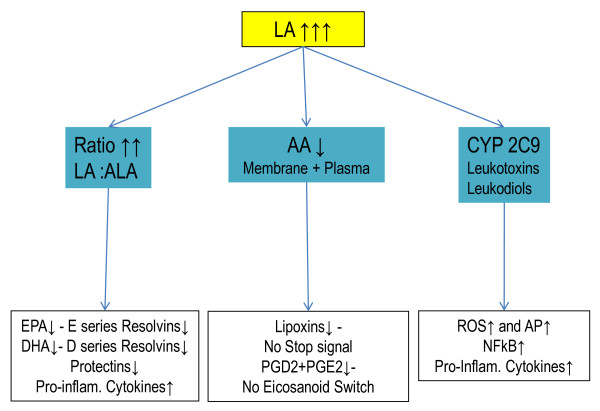
**Summary of the effects of high LA intake on Resoleomics**.

The intake ratio of α-linoleic acid (LA) (omega 6), α-linolenic acid (ALA) (omega 3), docosahexaenoic acid (DHA) and eicosapentaenoic acid (EPA) in the Western diet has changed dramatically compared to the estimated intake ratio of hunter-gatherer diets from 2-3:1 to 10-20:1 in the contemporary diet [42,43]. All of these PUFAs are essential for normal Resoleomics response, as they function as precursors for the special small mediators responsible for the instigation and conclusion of the inflammatory response. One of the toxic changes in fatty acid composition of food corresponds to the increased intake of LA since the production of vegetable oils in 1913. Increased LA levels affect the inflammation process in three ways (Figure 3):

1. Increase of the omega 6/omega 3 fatty acid ratio

2. Altered AA level

3. Increases of inflammatory compounds, leukotoxins (LK) production

#### Increased omega 6/omega 3 fatty acid ratio

**Figure 4:**
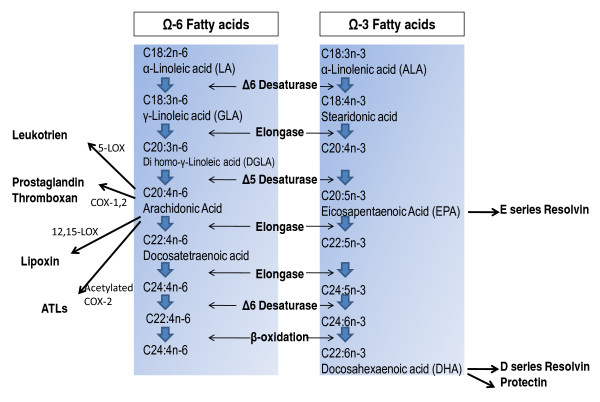
**Synthesis of unsaturated fatty acids in mammals by Desaturase and Elongase**.

The inflammatory effect of a high omega 6/omega 3 fatty acid ratio during inflammation has been demonstrated in recent human studies [44,45], in vitro studies [46,47] and animal studies [48,49]. The higher LA levels in phospholipids in plasma and cell membranes seem to be a major factor responsible for incomplete Resoleomics reactions. Higher intake of omega 3 fatty acids in the form of DHA and EPA regulate the production of pro-inflammatory cytokines and decrease LA levels in phospholipids in plasma and cell membranes [46,48]. The conversion of LA and ALA into respectively AA, DHA and EPA depend on the same enzymes in the desaturase and elongase cascade, with δ-6-desaturase as the rate-limiting enzyme (Figure 4) [50].

Human trials investigating the effects of omega 3 dietary supplements showed significant improvements of symptoms in patients suffering from diseases such as RA, inflammatory bowel disease, asthma, psoriasis, breast cancer and CVD. However, full remission of symptoms was not achieved [43,51]. Our conclusion is that an increased intake of omega 3 alone is not enough to restore Resoleomics; the intake of LA must be decreased as well.

#### LA effect on AA level

Higher AA levels in plasma result in more adequate inflammatory reactions, since AA is a precursor of pro- and anti-inflammatory substances within the self-limiting inflammatory process [52]. LA is the precursor for AA in the desaturase/elongase conversion (Figure 4). Theoretically, LA could be the source of a sufficient level of endogenous AA. However, higher intake of LA does not deliver increased levels of AA in comparison to low intake [53,54]. To achieve the required AA level, AA should be present in the regular diet [45]. The combined situation of AA deficiency together with a reduced intake of omega 3 fatty acids such as DHA and EPA (necessary for the flip flop reaction of LOX-5 and the Eicosanoid Switch [3]), enable a perpetuation of the pro-inflammatory initiation phase and therefore of chronic inflammation.

#### Increased production of leukotoxin

The third harmful effect of high LA intake is the possible production of so-called leukotoxins (LK). High LA levels are metabolized by CYP2C9 in the liver into biologically active oxidation products known as LK and leukotoxin diol (LTD). These metabolites promote oxidative stress responses and the activation of NFkB and AP-1, increasing the systemic release of pro-inflammatory cytokines [55]. LK and LTD are toxic for T cells, and can kill these cells with pathways resembling necrosis and programmed cell death [56].

### Role of high glycemic food in Inflammation

An abundant intake of high glycemic food appears to be related to an increased susceptibility to the development of chronic inflammation, as has been demonstrated by several research groups [57-59]. The consequences of a high carbohydrate diet are complex and multiple. The pathways leading to disturbances of normal inflammation are:

1. High glycemic food intake increases inflammation markers

2. High glycemic food intake causes hyperglycemia and hyperinsulinemia leading to disturbed balances in insulin growth factor-1 (IGF-1) and androgens

3. Chronic intake of high glycemic food causes hypoglycemia, which triggers central stress axes

#### High Glycemic food increases inflammation markers

Various clinical trials have shown that an abundant intake of high glycemic food increases inflammatory markers and markers of metabolic syndrome such as postprandial NFkB in mononuclear cells [57], high sensitive-C-Reactive Protein (hs-CRP)[58], interleukin (IL)-6, IL-7, IL-18 [60], levels of free radicals [59], cholesterol, triglycerides [61] and even blood pressure [62]. Changes incurred by following a low glycemic diet include improved insulin sensitivity, lower blood pressure and total cholesterol, which are all key markers of the metabolic syndrome [58,60,61]. The high glucose-induced inflammatory response is accompanied by hyperinsulinemia and insulin resistance, characteristic for people suffering from obesity [57,59]. Increased hsCRP values, hyperinsulinemia and insulin resistance are strongly related to CVD risk [60]. Glycemic index (GI) and glycemic load (GL) have therefore been proposed as biomarkers and predictors for (chronic) inflammation [63].

#### Hyperglycemia and hyperinsulinemia

**Figure 5:**
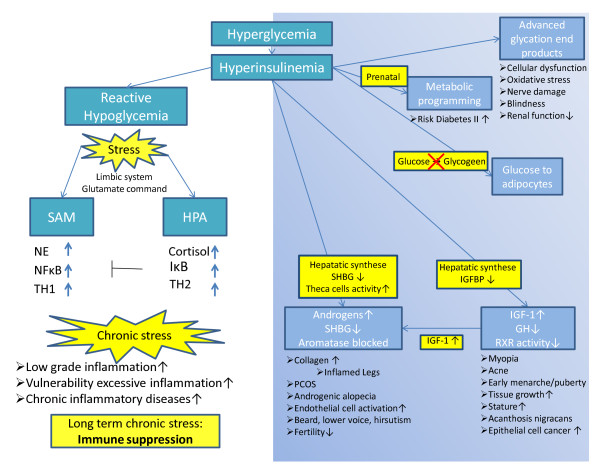
**High glycemic food intake could cause inflammation and diseases as a result of hyperinsulinemia**. The pathways in the shaded area have been extensively described by Cordain [64] (part B). Part A: The consequential reactive hyperglycemia is another deleterious pathway. Hyperglycemia is a danger signal, which activates the systemic stress system. Chronic activation will suppress the IIS, resulting in low grade inflammation and an increased vulnerability for excessive inflammation.

Cordain demonstrated that high glycemic food is a potential risk factor for inflammation through disturbed signalling of mechanisms as a result of hyperglycemia and hyperinsulinemia [64] (Figure 5b). Long exposure to high glucose levels in blood, which leads to a slow recovery of the homeostasis, makes tissues vulnerable to disease [65]. High plasma insulin can increase the production of IGF-1 and androgens. Both hormones are related to disorders such as polycystic ovarian syndrome (PCOS) [66], epithelial cell cancer (breast, prostate, colon) [67,68], acne [69], androgenic alopecia [70], and acanthosis nigricans [71]. Several pathways in this respect have been previously described in medical literature, but these go beyond the scope of this article.

#### Hypoglycemia triggers the systemic stress system

As previously mentioned, intake of a high glycemic diet can cause hyperglycemia and hyperinsulinemia. Hyperglycemia will push abundant glucose via insulin into muscle and adipocytes at the instigation of the inflammatory process. However, continuous intake of high glycemic food results in reactive hypoglycemia, ie an energy-deficient situation which threatens the homeostasis of the body. As a consequence, the brain will maintain its own energy supply aimed at the survival of the organism (the selfish brain) [25]. To ensure sufficient energy supply, the brain activates its systemic stress system to induce gluconeogenesis (Figure 5a). Excreted catecholamines and cortisol will mobilize extra energy, which is allocated with priority to the brain and to the activated IS, at the expense of other body tissues [72].

On the basis of the above information and other referenced data, it seems plausible to state that aspects of the Western diet, of the modern industrialised environment and of their resultant lifestyles form a chronic danger to the body, triggering both the central stress axes and the IIS into a state of chronic activity. This state seems to be a direct cause of the development of low-grade inflammation and consequently of chronic inflammatory diseases (Figure 5a).

## Impact of current medication on Resoleomics

The role of the IIS is to limit the damage of inflammation in acute situations. Anti-inflammatory medication can be used to dampen the immune response. Nowadays, as a result of lifestyle changes, man is exposed to chronic inflammation and consequently to the chronic use of anti-inflammatory medication, much of which in fact suppresses Resoleomics. Current medication used to treat chronic inflammatory diseases does suppress the symptoms of inflammation, but complete remission of the disease is seldom realized [73]. Resoleomics is hindered and complete resolution of the inflammation does not take place. Modern chronic inflammatory diseases are treated by several groups of medication. In this article we focus on rheumatoid arthritis (RA) medication as an example. Four groups of anti-inflammatory RA medication are taken into account: the prostaglandin inhibitors [Nonsteroidal anti-inflammatory drugs [NSAIDs: Aspirin (ASA) and COX-inhibitors], the Glucocorticoids (GCs), the Disease Modifying Drugs [DMARDs: Methotrexate (MTX) and Sulfasalazine (SSZ)] and the cytokine blockers [Biological agents: anti TNF-αlpha and IL-1 blockers]. The mechanisms of action and possible effects on the IIS and Resoleomics are summarized from literature (see Table 1). Most current therapies target the IIS in an attempt to inhibit the production of pro-inflammatory chemical mediators (Table 1). However, an equally important target is the active induction of pro-resolution programs by stromal cells such as fibroblasts within the inflamed tissues [74]. Inhibition of MIF [75] and production of NO [76] are not addressed in this article.

**Table 1:** Current RA treatments and their effect on immune system cells and predicted effect on Resoleomics

Medication	Mechanism of action	Current RA treatment effects on Immune System Cells	Predicted effects on ResoleomicsPhase 1: initiation, Phase 2: resolution, Phase 3: termination
Aspirin (ASA)[11,14,32,77-83]	COX-1 inhibition, COX-2 acetylationPGE2 ↓ATLs (15-epi-LX) ↑Activation of the ALX/FPR2 receptor ↑PLA2 ↓: free AA, PGE & LT ↓	PMN infiltration ↓, PGEs ↓, chemokines ↓Leucocyte accumulation ↓Neutrophil recruitment ↓Vascular permeability ↓Nonphlogistic phagocytosis of apoptoticneutrophils ↑	Negative: PG < LT levels: Phase 1 ↑Positive: PG not completely ↓: switch from phase 1 to phase 2 ↑Positive: ATLs ↑: phase 2 ↑ and 3 ↑
NSAIDs:COX-inhibitors [84,85]	COX-2 inhibition > COX-1 inhibitionPGE2 ↓, LTB4 ↑PGF2α, PGD2 ↓	COX-2 expression macrophages ↓:Chemotaxis of neutrophils, eosinophils and monocytes into synovium ↓	Negative: PG < LTB4 levels: Phase 1 ↑Switch from phase 1 to 2 ↓Switch from phase 2 to 3 ↓
Glucocorticosteroid (GCs)[32,80,86,87]	Transcription of IKB ↑: NFkB ↓Transcription by GCR &CREB-binding protein (CBP) ↓PLA2 ↓: free AA, PGE & LT ↓Annexin-1 ↑Activation of the ALX/FPR2 receptor ↑	PMN infiltration ↓, PGEs ↓, chemokines ↓Leucocyte accumulation ↓Neutrophil recruitment ↓NFkB - transcription ↓Expression of inflammatory genes ↓Macrophage migration and phagocytosis ↑	Negative: PGE, LT ↓: switch from phase 1 to 2 ↓Cortisol resistance: switch from phase 1 to phase 2 ↓ or no switchLipoxins ↓: switch from phase 2 to 3 ↓
DMARDs:Methotrexate MTX [88-102]	Folate analogs:1. Folate-dependent enzymes ↓:1a. Thymidylate synthetase1b. AICAR transformylase1c. Dihydrofolate reductase2. Cytosol peroxide (ROS) ↑	Ad 1a. Synthesis of DNA & RNA ↓T-cell- proliferation & protein- & cytokine-expression by T-cells ↓, LT & IL-1 ↓Ad 1b. Adenosine ↑: NK-cell, monocytes & macrophages functioning↓, Cytokine synthesis of TNF-α, IL-1, IL-6 & IL-8 ↓	
1c. THF ↓: purine & pyrimidine ↓Ad 2. T-cell apoptosis ↑	Negative: cytokines, T-cell activity ↓, LT ↓: switch from phase 1 to 2 ↓ or no switch		
DMARDs:Sulphasalazine (SSZ)[86]	SSZ: strong and potent inhibitor of NFkB-activation5-amino acytelate (5-ASA): PG ↓sulpha-pyridine	Less NFkB activation ↓: IL-2 of activated T-cells ↓, TNF alfa & IL-1 macro-phages ↓, Antibody in plasma cells ↓, Neutrophils, monocytes, macrohages, granulocyte activation ↓, IKB ↓: NFkB translocation ↓ & transcription of cytokines, adhesion molecules, chemokines ↓: COX-2 & PG↓	Negative: Immune cell activity ↓: switch from phase 1 to 2 ↓
Biological agents:Anti TNF-alpha [54,103-105]	TNF-alfa signalling of mono's, PMN's, T-cells, endothelial cells, synovial fibroblasts & adipocytes ↓COX-2 induction ↓	Monocyte activation, cytokine & PG release ↓PMN priming, apoptosis and oxidative burst; T-cell apoptosis, clonal regulation & T-cell receptor ↓Endothelial-cell adhesion molecule expression, cytokine release ↓synovial fibroblast proliferation, collagen synthesis, MMP & cytokine release ↓Adipocyte FFA release ↑	Negative: Immune cell activity ↓: switch from phase 1 to 2 ↓
Biological agents:IL-1 blocker [54,103-105]	IL-1 signalling of monocytes, B-cells, endothelial cell, synovial fibroblasts, chrondrocytes ↓COX-2 induction ↓	Synovial fibroblast cytokine, chemokine, MMP, iNOS & PG release ↓Mono's cytokine, ROI & PG release ↓Osteoclast activity ↑GAG synthesis ↓, iNOS ↑, MMP & aggrecanaseEndothelial-cell adhesion molecule expression↓	Negative: Immune cell activity ↓: switch from phase 1 to 2 ↓

### Positive effect of ASA and GCs on Resoleomics

Medical intervention should stimulate the endogenous pathways of resolution and two drugs already known to possess these qualities are central to contemporary medicine: glucocorticoids (GCs) [77] and aspirin (ASA) [106,107]. It is apparent that ASA and GCs have a positive effect on Resoleomics, while other medications prolong the initiation phase, tempering and/or blocking the resolution and termination phase of Resoleomics in various ways (Table 1). The positive effect of ASA on Resoleomics can be ascribed to its ability to produce ASA-triggered lipoxins (ATLs) through acetylation (and not through an irreversible inhibition) of the COX-2 enzymes [78]. These ATLs show many pro-resolving properties, which are essential in the resolution and termination phase of the inflammation process [79,108]. Long-term intake of high doses of ASA blocks PGE2 production and initiates the resolution phase without affecting the biosynthesis of other pro-resolving mediators [108]. Low and high doses of ASA increase the production of lipoxin A4 (LXA4) and 15-epi-LXA4 in the rat brain, suggesting that ASA could protect against neuroinflammation [109]. However, because of its side effects, ASA is no longer the treatment of choice for RA. In high doses, inhibition of the COX-1 enzyme by ASA is responsible for damage to the stomach lining.

ASA and also GCs activate the ALX/FRP2 receptor, making them the ideal collaborator in the resolution process [77]. GCs-induced annexin-1 protein (ANXA1) [110,111] as well as ASA-induced ATLs act on the same ALX/FPR2 receptor and dampen PMN infiltration [77,80]. ANXA1 also inhibits the phospholipids A2 enzyme (PLA2). Reduced PLA2 activity appears to reduce AA release from the cell membrane [32,112], which possibly leads to decreased levels of both PGs and LTs and to the delay of resolution. Besides their anti-inflammatory effects, GCs have a positive influence on resolution by enhancing macrophage migration and phagocytosis [11,113].

### Adverse effects of medication on Resoleomics

The use of anti-inflammatory medication without the capacity to induce (complete) resolution should be considered solution-toxic, ie hindering Resoleomics. NSAIDs are strong inhibitors of COX-2 and less of COX-1 enzymes [114]. Almost complete COX-2 inhibition decreases the PGs synthesis, and consequently leads to a higher production of LTs via LOX-5 in PMNs [115]. PGE2 and PgD2 decrease the activity of LOX-5, decreasing neutrophil activity and facilitating the end of the inflammatory phase and the instigation of resolution.

Immune-suppressors such as SSZ (and less powerful GCs) almost completely block NF-kB transcription, leading to insufficient cytokine production and suboptimal inflammation [86]. Again the resolution process will not be completed, with perpetuation of inflammation as the logical consequence.

Perhaps the most deleterious drugs, interfering negatively with resolution, are TNF-alpha inhibitors such as anti TNF-alpha and MTX. MTX inhibits the proliferation of the IIS cells, decreasing the production and accumulation of adenosine within the IS cells [88,116]. These effects lead to rapid anti-inflammatory effects and symptom release. However, because of its side effects and incomplete resolution, this medication is qualified as solution-toxic. This conclusion is supported by many patients who have discontinued this treatment [73].

Another group of possible solution-toxic drugs are biological agents with an inhibiting effect on TNF-alpha and IL-1. Biological agents together with DMARDS (Table 1) are strong anti-inflammatory compounds, decreasing the production of pro-inflammatory cytokines. The absence or insufficient activity of pro-inflammatory cytokines decreases cell communication and induction of COX-2 in activated neutrophils. This can lead to less production of resolution substances such as PgE2, PgD2 and lipoxins [54,103]. Furthermore, DMARDs and biological agents appear to reduce the functioning and number of IIS cells, causing suboptimal inflammation and possibly inflammation perpetuation [104].

## Discussion

Long-term activity of the IIS results in low-grade inflammation and chronic disease. Over the past years, ideas regarding the treatment of inflammation have started to change as evidence accumulates which shows that, although the targeting of infiltrating immune cells can control the inflammatory response, it does not lead to its complete resolution and a return to homeostasis, which is essential for healthy tissue and good health in general.

Hotamisligil describes how low-grade, chronic inflammation ('meta-inflammation') induced by a nutritional and metabolic surplus, is accompanied by disturbed metabolic pathways and chronic metabolic disorders. He states that this inflammatory response differs from the classical inflammation response caused by injury [117]. However, others have shown that the classical response of the IIS dealing with injuries can be linked to activation of the central stress axes [26,28]. This article specifically discusses the relationship between the over-activated systemic stress system and the self-limited process of inflammation, known as Resoleomics, executed and controlled by the innate immune system (IIS).

Changes in lifestyle which are new to our evolutionary process should be considered a major trigger in causing chronic activation of the IS and consequently of the central stress axes and vice versa, thereby leading to chronic diseases such as cardiovascular diseases (CVD), diabetes, respiratory diseases, mental disorders, auto-immune diseases (AID) and cancers. This article evaluates two of the lifestyle changes which contribute to long-term activity of the ISS, namely, nutrition and continuous psycho-emotional stress. Other risk factors such as physical inactivity [6], genetic susceptibility [118], smoking, environmental toxicity and shift work [119] fall beyond the scope of this article but should not be ruled out.

**Figure 6:**
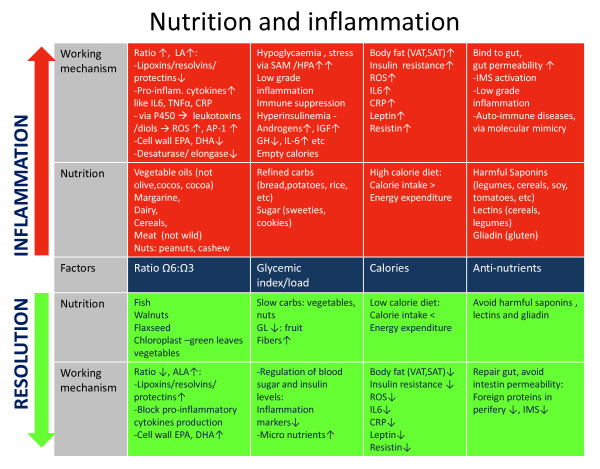
**Reflection of the working mechanism demonstrating how several nutritional factors could induce and inhibit inflammation**.

Nutrition is an important factor in understanding the development of chronic inflammation. The current Western diet can disturb the resolution response in various ways (Figure 6). In the Ancestral human diet, foodstuffs with an increased risk of inflammation were virtually unknown, while nutrients able to activate the IIS are now abundant in our diet [38,120]. Cordain's research has focused on relating these anti-nutrients in food (eg lectines, saponines) to the development of chronic inflammation and autoimmune diseases (AID) [7,39]. Fortunately, it seems that the human body possesses a strong capacity to recover from illness. If our genes are exposed to their 'original' environment by intake of an ancestral human diet, their function can recover rapidly. Research has shown that obese persons improve their blood markers after just 10 days following a paleolithic diet consisting of fish, lean meat, fruit, vegetables and nuts [121]. Similar results have been found in a study with aboriginals suffering from Diabetes II, who showed normalized blood markers after returning to their traditional lifestyle for seven weeks [122].

People suffering from chronic inflammatory disease demonstrate over-activated central stress axes, which then lead to catecholamines, cortisol and insulin resistance. McGowan et al [123] show the impact of childhood abuse on the epigenetic pattern of different genes including the gene for GR in the hippocampus. They found a decreased level of GR and an increased methylation pattern of the GR gene, giving rise to a situation of lower cortisol sensibility and altered HPA stress responses. This could make people more vulnerable to developing diseases. An altered sensitivity to cortisol has been linked to diseases such as rheumatoid arthritis (RA) [124], post-traumatic stress syndrome [125], chronic fatigue syndrome [126], inflammatory diseases and AID in general [127].

**Figure 7:**
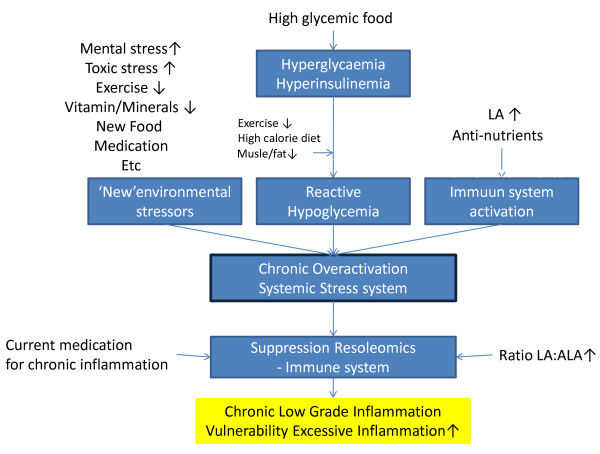
**Chronic over-activation of the systemic stress system as a result of external stressors plays a central role in the development of chronic inflammatory diseases**. Current intervention with anti-inflammatory medication suppresses Resoleomics and the IIS and so enhance the over-activation of the systemic stress system.

The key priority in the treatment of people with chronic inflammation is to induce the Eicosanoid Switch to the anti-inflammatory resolution phase. Long-lasting cortisol resistance and insulin resistance will definitely delay or block complete resolution. The combination of local factors (ie DHA deficiency, low levels of protectins) disturbing the process of complete resolution (ie Resoleomics) and the absence of adequate NE and cortisol signalling can be responsible for perpetuatual inflammation by delaying the resolution phase of the inflammatory response (Figure 7).

Current anti-inflammatory medication used in RA treatment is aimed at the suppression of the IIS and its inflammatory response and thus hinders Resoleomics. In addition, these medication interventions do not solve underlying catecholamine, cortisol and insulin resistance, and consequently making it impossible to achieve full recovery of the chronic inflammation. This suggests that chronic use of anti-inflammatory medication in fact impedes the body from making a full recovery. Furthermore, the ongoing low-grade inflammation will continuously trigger the activity of the systemic stress system [28].

Health care should focus on early detection of silent, ongoing and low-grade inflammation in order to avoid the development of many chronic diseases. Further research is needed to validate a questionnaire which addresses early symptoms of chronic low-grade inflammation, ie avoidance of exercise, fatigue, emotional flatness, social isolation, decreased libido, hyper or hyposomnia, obsessive behaviour or sensitivity to addiction [6,128].

We have made an effort to demonstrate that the science of Resoleomics can help to find new ways to treat people suffering from diseases based on chronic inflammation. Since over-activated central stress axes directly delay Resoleomics, and thereby delay the resolution of inflammation, treatment should focus on restoring the central stress system to its default, healthy homeostasis. Dietary changes, psycho-emotional stress release and physical activity should always be included in treatment of all chronic inflammatory diseases.

## Abbreviations

AA: Arachidonic acid; Ach: Acetylcholine; AID: Autoimmune diseases; ALA: α-linolenic acid; ALX/FPR2: Lipoxin A(4) receptor; ANXA 1: Annexin 1 protein; AP-1: Activator protein 1; ASA: Aspirin; ATLs: Stable aspirin-triggered lipoxin; COX: Cyclo-oygenase; CRP: High sensitive-C- Reactive Protein; CVD: Cardiovascular diseases; DHA: Docosahexaenoic acid; DMARDs: Disease Modifying Drugs; EPA: Eicosapentaenoic acid; GI, Glycemic index; GL: Glycemic load; GCs: Glucocorticoids; HPA: Hypothalamus-pituitary-adrenal; IGF-1: Insulin growth factor-1; IS: Immune system; IIS: Innate immune system; IL: Interleukin; LA: α-linoleic acid; LC: Locus Coeruleus; LOX: Lipoxygenase; LK: Leukotoxins; LTs: Leukotrienes; LTD: Leukotoxin diol; LXs: Lipoxins; MTX: Methotrexate; NE: Norepinephrine (ie noradrenaline); NF-kB: Nuclear factor-KappaB; NSAIDs: Nonsteroidal anti-inflammatory drugs; PCOS: Polycystic ovarian syndrome; PGs/PGE2/PGD2/PGF2a: Prostaglandins/prostaglandin E2, D2, F2a; PLA2: Phospholipase A2 enzyme; PMNs: Polymorphonuclear leukocytes; PUFAs: Polyunsaturated fatty acids; RA: Rheumatoid arthritis; SAM: Sympathetic-adrenal-medulla; SFA: Saturated fatty acids; SNS: Sympathetic nervous system; TNF: Tumour necrosis factor.

## Competing interests

The authors declare that they have no competing interests.

## Authors' contributions

MMB executed an analysis and review of the relationship between chronic inflammatory pathways and the central stress systems, Resoleomics and nutrition. MMB also drafted the manuscript. MLvW reviewed the MOA of currently used anti-inflammatory medication and its effect on Resoleomics. LP played a central role in integrating the results of various stressors on chronic inflammation pathways and also acted as lead reviewer. All authors have approved the final manuscript.

## Authors' information

MMB and MLvW, MD treat patients with chronic diseases in a private practice. LP, a practising psychoneuroimmunologist and associate Professor at the University of Gerona, Spain, has developed valuable insights into the metabolic pathways of chronic diseases, which he has applied in the treatment of numerous patients.
